# Complete Genome Sequence of *Lactobacillus casei* LC5, a Potential Probiotics for Atopic Dermatitis

**DOI:** 10.3389/fimmu.2017.00413

**Published:** 2017-04-07

**Authors:** Jisu Kang, Won-Hyong Chung, Tae-Joong Lim, Tae Woong Whon, Sanghyun Lim, Young-Do Nam

**Affiliations:** ^1^Research Group of Gut Microbiome, Korea Food Research Institute, Sungnam, South Korea; ^2^Department of Food Biotechnology, Korea University of Science and Technology, Daejeon, South Korea; ^3^Research and Development Center, Cell Biotech Co. Ltd., Gimpo, South Korea; ^4^Department of Biology, Kyung Hee University, Seoul, South Korea

**Keywords:** atopic dermatitis, probiotics, *Lactobacillus casei*, genome sequence, PacBio

## Background

Probiotics are living microorganisms providing health beneficial effect to the host (1). Probiotics have been used for the treatment or prevention of various diseases related to diarrhea (2), cholesterol (3) immune function (4), and inflammatory bowel disease (5). In addition, recent study also presents that probiotic bacteria in the *Bifidobacterium* and *Lactobacillus* genera are able to have therapeutic effects in the patients of psychological disorders, such as depression, anxiety, and memory (6).

*Lactobacillus casei* is a Gram-positive bacterium that naturally inhabits the human and animal gastrointestinal and mouth organs (7). As its name implies, this heterofermentative microorganism is the dominant species present in ripening cheddar cheese (8). In probiotic aspects, *L. casei* showed beneficial roles in the activation of the gut mucosal immune system (9), treatment of diabetics (10), and chronic constipation (11). In the previous study, we isolated *L. casei* LC5 strain from fermented dairy products, which showed immune regulatory functions, especially, therapeutic effect on atopic dermatitis as a member of complex probiotics (12–14).

In order to gain better insight of the probiotic effect on atopic dermatitis, we analyzed the genome sequence of *L. casei* LC5. According to the report of NCBI Genome,^1^ more than two hundreds of *Lactobacillus* organisms are sequenced and their beneficial properties derived from genomic information are used in the food industry. However, the available genomes of *L. casei* strains as members of health promoting probiotics are still insufficient. Furthermore, *L. casei* strains are frequently confused with the closely related strains such as *Lactobacillus paracasei* and *Lactobacillus rhamnosus*. Therefore, comparative study in a whole genome scale is required to clarify taxonomic association of *L. casei* LC5 as well as its functional characteristics. The availability of the genomic information of *L. casei* LC5 will aid as a basis for further in-depth analysis of the probiotic function of *L. casei* strains.

## Materials and Methods

### Bacterial Strains and DNA Preparation

*Lactobacillus casei* LC5 was isolated from fermented dairy products and commercially used as probiotics in Korea (15). *L. casei* LC5 was cultured aerobically in MRS medium (Difco, USA) at 37°C for 18 h. Genomic DNA from *L. casei* LC5 was extracted and purified using a QIAamp DNA Mini Kit (Qiagen, Germany). The concentration of genomic DNA was qualified with NanoDrop 2000 UV–vis spectrophotometer (Thermo Scientific, USA) and Qubit 2.0 fluorometer (Life Technology, USA).

### Genome Sequencing, Assembly, and Annotation

Whole genome sequencing of *L. casei* LC5 was carried out by using PacBio RS II platform. A 20 kb DNA library was constructed according to the manufacturer’s instruction and sequenced using single molecule real-time (SMRT) sequencing technology with the P6 DNA polymerase and C4 chemistry. A total of 138,180 subreads (1.04 Gb) were obtained with 400-fold coverage. The average length of subreads was 7,550 bp and N50 was 10,940 bp. Genome assembly was performed using HGAP 3.0 (16) with default options. The annotation was carried out with NCBI Prokaryotic Genome Annotation Pipeline (17) through NCBI Genome submission portal (GenomeSubmit at http://ncbi.nlm.nih.gov). The chromosome topology was drawn using DNAPlotter (18). Clusters of orthologous groups (COG) categories were assigned to the coding genes using BLASTP (e-value: 1e−3) against COG database (19).

### Phylogenetic Analysis and Comparative Genomic Analysis

For phylogenetic and comparative study, we downloaded 19 genome sequences of *L. casei* group (10 of *L. casei*, 8 of *L. paracasei*, 1 of *Lactobacillus zeae*, and 1 of *L. rhamnosus*) from NCBI genome database.^2^ A list of the reference genomes are as follows: *L. casei* Zhang (NC_014334), *L. casei* BL23 (NC_010999), *L. casei* BD-II (NC_017474), *L. casei* LC2W (NC_017473), *L. casei* 12A (NZ_CP006690), *L. casei* W56 (NC_018641), *L. casei* LcY (NZ_CM001848), *L. casei* LcA (NZ_CM001861), *L. casei* LOCK919 (NC_021721), *L. casei* ATCC 393 (NZ_AP012544), *L. paracasei* ATCC 334 (NC_008526), *L. paracasei* 362.5013889 (NC_022112), *L. paracasei* N1115 (NZ_CP007122), *L. paracasei* JCM (NZ_AP012541), *L. paracasei* CAUH35 (NZ_CP012187), *L. paracasei* L9 (NZ_CP012148), *L. paracasei* KL1 (NZ_CP013921), *L. zeae* DSM 20178 (NZ_AZCT01000001), and *L. rhamnosus* GG (NC_013198). The assembly levels of all genomes are “complete genome” or chromosome except *L. zeae* DSM 20178 (includes 55 scaffolds). Because we failed to fetch full-length 16S rRNA gene from the genome of *L. zeae* DSM 20178, we alternatively used a 16S rRNA gene of *L. zeae* RIA 482 (NR_037122), the closest sequence of DSM 20178 (sequence identity = 99.9%), in the phylogenetic analysis.

The evolutionary history was inferred by using the maximum likelihood method based on the Tamura–Nei model (20). All positions containing gaps and missing data were eliminated. There were a total of 1521 positions in the final dataset. Those phylogenetic analyses were conducted in MEGA6 (21). To compute genomic distance, we first computed orthologous average nucleotide identity (OrthoANI) values using orthologous average nucleotide identity tool (22). The OrthoANI values were converted to distance values by following formula: distance = 1 − (OrthoANI/100). The evolutionary distance was computed using the neighbor-joining method of MEGA6 (21). The tree is drawn to scale with branch lengths in the same units as those of the evolutionary distances used to infer the phylogenetic tree. The resulting phylogenetic tree was produced using MEGA6. Pan-genomic study using Panseq (23) was performed to investigate the genomic conservation and finding novel region in the sequenced genome.

## Results

### Genome Characteristics of *L. casei* LC5

**Figure 1:**
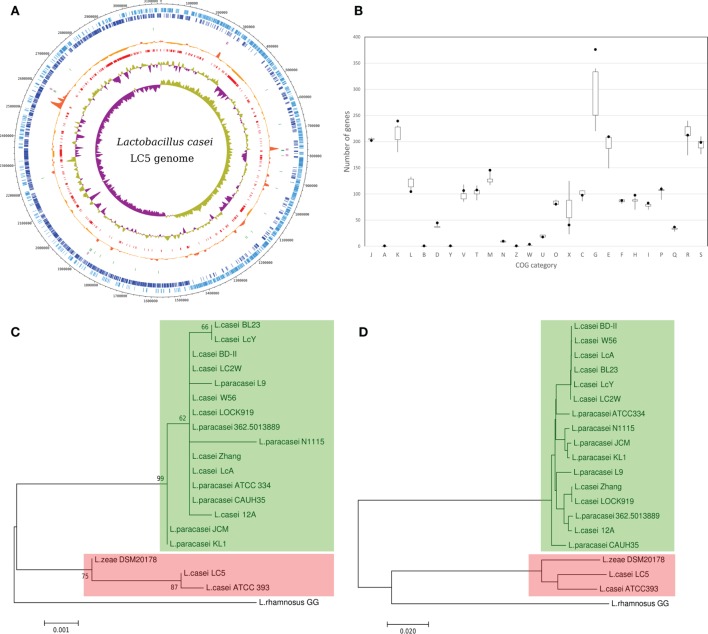
**Genome characteristics of *Lactobacillus casei* LC5 genome**. **(A)** Circular map of genomic features; eight tracks were plotted in the map. Track 1 (light blue; outermost), forward-stranded coding CDS; Track 2 (blue), reverse-stranded coding CDS; Track 3 (light purple), rRNA including 5S, 16S, and 23S; Track 4 (green), Trna; Track 5 (orange), peak of pan-genomic conservation; Track 6 (red), novel regions (below 85% similarities with the other genomes); Track 7 (light green and purple), GC content; and Track 8 (light green and purple), GC skew. **(B)** Abundance of clusters of orthologous groups (COG) categories; black point indicates the abundance of LC5 for each category. A box and whisker plot indicates a statistical distribution of the COG categories of 19 *L. casei* genomes. **(C)** Phylogenetic tree of *L. casei* group based on 16S rRNA genes and **(D)** phylogenetic tree of *L. casei* group based on average nucleotide identity. The value 0.02 of the ruler in **(D)** indicates 2% of genomic dissimilarity. Red boxes on the **(C,D)** indicate the genomes associated to the high-GC group and green boxes indicate the genomes associated to the low-GC group.

We obtained a complete genome sequence of *L. casei* LC5 using SMRT sequencing. This genome has a chromosome and no organelle sequences. The total size of the genome is 3,132,867 bp and its GC content is 47.9%. A total of 2,925 genes were detected from the genome sequence. The number of coding CDS is 2,817 and pseudogenes is 31. Seventy seven RNAs (15 rRNAs, 59 tRNAs, and 3 non-coding RNAs) were also identified. Repeating region or CRISPR array was not identified. Genomic features of *L. casei* LC5 are shown in Figure 1A.

Although *L. casei* LC5 was identified as a strain of *L. casei*, it showed different genomic features compared to the other published *L. casei* strains; According to the summary of 37 *L. casei* genomes deposited in NCBI Assembly, the median length is 3.01993 Mb, the median of coding genes is 2,712, and the median of GC contents is 46.4%. An interesting point is that those genomes can be split into two groups by the difference of GC contents, high-GC group (47.7–47.9%) and low-GC group (46.2–46.6%). Five genomes (ATCC 393, N87, 867_LCAS, Lbs2, JCM 1134) and *L. casei* LC5 belong to the high-GC group and the other genomes belong to the low-GC group (Table 1).

**Table 1:** **Genome summary of *Lactobacillus casei* group**.

Organism/name	Strain	Clade	Assembly level	Size (Mb)	GC%	GC group
*L. casei* LC5	LC5	*L. casei*	Complete genome	3.13	47.9	High
*L. case*i str. Zhang	Zhang	*L. casei*	Complete genome	2.90	46.4	Low
*L. casei* BL23	BL23	*L. casei*	Complete genome	3.08	46.3	Low
*L. casei* BD-II	BD-II	*L. casei*	complete genome	3.13	46.3	Low
*L. casei* LC2W	LC2W	*L. casei*	Complete genome	3.08	46.4	Low
*L. casei* 12A	12A	*L. casei*	Complete genome	2.91	46.4	Low
*L. casei* W56	W56	*L. casei*	Complete genome	3.13	46.3	Low
*L. casei* LOCK919	LOCK919	*L. casei*	Complete genome	3.14	46.2	Low
*L. casei* subsp. casei ATCC 393	ATCC 393	*L. casei*	Complete genome	2.95	47.9	High
*L. casei* LcY	LcY	*L. casei*	Chromosome	3.10	46.3	Low
*L. casei* LcA	LcA	*L. casei*	Chromosome	3.13	46.3	Low
*L. casei* A2-362	A2-362	*L. casei*	Scaffold	3.19	46.2	Low
*L. casei*	KL1-Liu	*L. casei*	Scaffold	2.85	46.6	Low
*L. casei* DSM 20011 = JCM 1134	DSM 20011	*L. casei*	Scaffold	2.82	46.5	Low
*L. casei* 21/1	21/1	*L. casei*	Contig	3.22	46.2	Low
*L. casei* 32G	32G	*L. casei*	Contig	3.01	46.4	Low
*L. casei* A2-362	A2-362	*L. casei*	Contig	3.36	46.1	Low
*L. casei* CRF28	CRF28	*L. casei*	Contig	3.04	46.3	Low
*L. casei* M36	M36	*L. casei*	Contig	3.15	46.3	Low
*L. casei* T71499	T71499	*L. casei*	Contig	3.00	46.2	Low
*L. casei* UCD174	UCD174	*L. casei*	Contig	3.07	46.4	Low
*L. casei* UW1	UW1	*L. casei*	Contig	2.87	46.4	Low
*L. casei* UW4	UW4	*L. casei*	Contig	2.76	46.4	Low
*L. casei* Lc-10	Lc-10	*L. casei*	Contig	2.95	46.4	Low
*L. casei* Lpc-37	Lpc-37	*L. casei*	Contig	3.08	46.3	Low
*L. casei* UW4	UW4	*L. casei*	Contig	2.63	46.4	Low
*L. casei* 12A	12A	*L. casei*	Contig	2.93	46.3	Low
*L. casei* 5b	5b	*L. casei*	Contig	3.02	46.3	Low
*L. casei*	N87	*L. casei*	Contig	3.00	47.9	High
*L. casei*	867_LCAS	*L. casei*	Contig	3.09	47.9	High
*L. casei*	DPC6800	*L. casei*	Contig	3.05	46.4	Low
*L. casei*	Lc1542	*L. casei*	Contig	2.92	46.5	Low
*L. casei*	1316.rep1_LPAR	*L. casei*	Scaffold	2.86	46.5	Low
*L. casei*	1316.rep2_LPAR	*L. casei*	Scaffold	2.79	46.4	Low
*L. casei*	844_LCAS	*L. casei*	Scaffold	2.79	46.4	Low
*L. casei*	BM-LC14617	*L. casei*	Scaffold	3.04	46.3	Low
*L. casei*	Lbs2	*L. casei*	Scaffold	3.27	47.9	High
*L. casei* DSM 20011 = JCM 1134	JCM 1134	*L. casei*	Contig	2.78	47.7	High
*Lactobacillus paracasei* ATCC 334	ATCC 334	*L. paracasei*	Complete genome	2.92	46.6	Low
*L. paracasei* subsp. *paracasei* 8700:2	8700:2	*L. paracasei*	Complete genome	3.03	46.3	Low
*L. paracasei* N1115	N1115	*L. paracasei*	Complete genome	3.06	46.5	Low
*L. paracasei subsp. paracasei* JCM 8130	JCM 8130	*L. paracasei*	Complete genome	3.02	46.6	Low
*L. paracasei*	CAUH35	*L. paracasei*	Complete genome	2.97	46.3	Low
*L. paracasei*	L9	*L. paracasei*	Complete genome	3.08	46.3	Low
*L. paracasei*	KL1	*L. paracasei*	Complete genome	2.92	46.6	Low
*Lactobacillus zeae* DSM 20178 = KCTC 3804	DSM 20178	*L. zeae*	Scaffold	3.12	47.7	High
*Lactobacillus rhamnosus* GG	GG (ATCC 53103)	*L. rhamnosus*	Complete genome	3.01	46.7	Low

### Comparative Study of *L. casei* Group

Comparative study of both 16S rRNA genes and whole genome sequences revealed that the closest genome of *L. casei* LC5 was *L. casei* ATCC 393 and second closest one was *L. zeae* DSM 20178. The three genomes which showed distinguishable differences on the comparative study, LC5, ATCC 393, and *L. zeae* DSM 20178, belong to the high-GC group as described in the above section. In contrast to the phylogenetic distances based on 16S rRNA gene among the high-GC group (below 0.001), the distances between the high-GC group and the low-GC group were above 0.003 (Figure 1C). It was also supported by the estimation result of the whole genomic comparison. Average nucleotide identity (ANI) values among the high-GC group were above 94% whereas ANI values between two groups were below 80% (Figure 1D). All the *L. casei* strains and *L. paracasei* strains belonging to the low-GC group showed the high genomic similarity of 98% or higher.

### Functional Classification

Functional classification based on COG assigned the 2,334 CDSs into the 1,309 COG numbers. From the comparison of functional categories against the 19 *L. casei* group genomes, we found that *L. casei* LC5 contains the high number of proteins which associate with “carbohydrate transport and metabolism (G)” (376 proteins) and “transcription (K)” (239 proteins) excluding two unknown categories, “general function prediction only (R)” and “function unknown (S)” as shown in Figure 1B. *L. casei* LC5 has at least 36 more proteins than the other genomes on the category G and has at least 8 more proteins than the other genomes on the category K. The gene expansion of those two functional categories in the LC5 genome is not found on the other members of high-GC group. Although the genomes belonging to high-GC group showed high similarities to each other and the genomes belonging to the high-GC group do not have excessive proteins on the categories, G and K, when compared to those belonging to the low-GC group. Moreover, *L. casei* ATCC 393 which is the most similar genome of LC5 has fewer proteins than the average number of those categories, 223 proteins for the category G and 192 proteins for the category K.

In the previous study, probiotic LC5 strain isolated from Korean fermented dairy product showed great therapeutic effect on atopic dermatitis. Here, we report a genomic overview and distinguishing gene features of LC5 by comparative genomic analysis of 20 related strains. The genomic data presented in this report will broaden our knowledge about roles and mechanisms of microorganisms ameliorating symptoms of immune diseases and help developing functional probiotics for the treatment of immune disorders.

## Data Access

The *L. casei* LC5 genome sequencing project has been deposited at GenBank under the accession number CP017065. The BioProject and BioSample designation for this project is PRJNA340077 and SAMN05631198, respectively. This strain has been deposited in the Korean Collection for Type Cultures (deposit ID: KCTC 12398BP).

## Author Contributions

Y-DN and SL designed and coordinated all the experiments. T-JL and JK performed cultivation and DNA preparation. JK and W-HC performed genome assembly, gene prediction, gene annotation, and comparative genomic analysis. Y-DN, W-HC, TW, and JK wrote the manuscript. All authors have read the manuscript and approved.

## Conflict of Interest Statement

The authors declare that the research was conducted in the absence of any commercial or financial relationships that could be construed as a potential conflict of interest.

## Footnotes

^1^https://www.ncbi.nlm.nih.gov/genome/?term=Lactobacillus.

^2^http://www.ncbi.nlm.nih.gov/genome/.
